# Expression of Serum Retinol Binding Protein and Transthyretin within Mouse Gastric Ghrelin Cells

**DOI:** 10.1371/journal.pone.0064882

**Published:** 2013-06-19

**Authors:** Angela K. Walker, Zhi Gong, Won-Mee Park, Jeffrey M. Zigman, Ichiro Sakata

**Affiliations:** 1 Departments of Internal Medicine (Divisions of Hypothalamic Research and Endocrinology & Metabolism) and Psychiatry, the University of Texas Southwestern Medical Center, Dallas, Texas, United States of America; 2 Area of Regulatory Biology, Division of Life Science, Graduate School of Science and Engineering, Saitama University, Saitama, Japan; University of Cordoba, Spain

## Abstract

Ghrelin is an orexigenic peptide hormone produced mainly by a distinct group of dispersed endocrine cells located within the gastric oxyntic mucosa. Besides secreted gene products derived from the preproghrelin gene, which include acyl-ghrelin, desacyl-ghrelin and obestatin, ghrelin cells also synthesize the secreted protein nesfatin-1. The main goal of the current study was to identify other proteins secreted from ghrelin cells. An initial gene chip screen using mRNAs derived from highly enriched pools of mouse gastric ghrelin cells demonstrated high levels of serum retinol-binding protein (RBP4) and transthyretin (TTR), both of which are known to circulate in the bloodstream bound to each other. This high expression was confirmed by quantitative RT-PCR using as template mRNA derived from the enriched gastric ghrelin cell pools and from two ghrelin-producing cell lines (SG-1 and PG-1). RBP4 protein also was shown to be secreted into the culture medium of ghrelin cell lines. Neither acute nor chronic caloric restriction had a significant effect on RBP4 mRNA levels within stomachs of C57BL/6J mice, although both manipulations significantly decreased stomach TTR mRNA levels. In vitro studies using PG-1 cells showed no effect on RBP4 release of octanoic acid, epinephrine or norepinephrine, all of which are known to act directly on ghrelin cells to stimulate ghrelin secretion. These data provide new insights into ghrelin cell physiology, and given the known functions of RBP4 and TTR, support an emerging role for the ghrelin cell in blood glucose handling and metabolism.

## Introduction

The gastrointestinal tract is home to numerous endocrine cell types, the hormonal products of which play critical roles in several physiologic processes and behaviors, including eating, energy homeostasis, glucose metabolism, and gastrointestinal motility [1], [2]. Ghrelin is one such gastrointestinal product that is unique in that it is the only known peptide hormone produced in the periphery that potently stimulates food intake [3]. Ghrelin is also unique in that it is the only known peptide to be post-translationally modified by *O*-acylation with an octanoic acid group, which is essential for ghrelin binding to its only identified receptor, GHSR (growth hormone secretagogue receptor) [3], [4], [5]. Besides enhancing food intake, ghrelin also regulates growth hormone secretion, several food reward behaviors, body weight, blood glucose balance, gastrointestinal prokinesis and mood-related behaviors, among many other actions [6], [7], [8], [9], [10], [11], [12], [13], [14], [15], [16], [17].

Ghrelin expression is found throughout the gastrointestinal tract, although most ghrelin circulating in the bloodstream is thought to emanate from a distinct group of sparsely distributed gastric mucosal ghrelin cells [3], [18], [19], [20]. Prior to the discovery of ghrelin, these cells had been identifiable due to their characteristic round, compact, electron-dense secretory granules [18], [19], [21], [22]. Using mRNA derived from enriched pools of ghrelin cells [isolated from transgenic mice which express humanized *Renilla reniformis* green fluorescent protein (hrGFP) under the control of the ghrelin promoter], our group has described the expression of chromogranin A and prohormone convertases 1/3 and 2 within gastric ghrelin cells, thus confirming the peptide hormone-producing nature of these cells [23]. Similar types of quantitative RT-PCR (qPCR) analyses on fluorescence activated cell sorting (FACS)-separated, GFP-labeled ghrelin cells have been used to determine the membrane-bound *O*-acyltransferase and adrenergic receptor expression profile of ghrelin cells, as well as their expression of several enzymes, receptors, and channels that potentially help mediate the response of ghrelin cells to D-glucose and other hormones [24], [25], [26], [27]. A combination of qPCR analyses and cell culture techniques using immortalized ghrelin cell lines derived from transgenic mice containing gastric ghrelinomas has helped establish the β_1_-adrenergic receptor system as a key regulator of ghrelin biosynthesis and secretion [25], [28]. Ghrelin secretion in response to direct exposure of immortalized gastric ghrelin cell lines to a variety of other peptide hormones, monoamines, and other compounds also has been investigated, with a resulting inhibitory or null effect described for insulin and somatostatin and a stimulatory effect identified for dopamine and oxytocin [25], [28], [29]. Many of these findings have been reproduced in ghrelin cell-containing primary cultures of dispersed mouse or rat gastric mucosal cells and in primary cultures of highly purified pools of gastric ghrelin cells [26], [27], [30], [31], [32]. Much remains to be discovered regarding ghrelin cell physiology and pathophysiology.

Accumulating evidence suggests that gastric ghrelin cells synthesize more than just one secreted product [33]. Both acyl- and desacyl-forms of ghrelin are presumed to be released from ghrelin cells [25], [26], [34], [35]. In addition, and similar to enteroendocrine L-cells which secrete multiple peptide hormones resulting from prohormone convertase-mediated cleavage of the preproglucagon gene, ghrelin cells secrete at least two gene products derived from prohormone convertase 1/3-mediated cleavage of the preproghrelin gene: ghrelin and obestatin [36], [37], [38]. Obestatin is a 23-amino acid peptide that was originally reported to have effects on food intake and gastrointestinal motility opposite to those of acyl-ghrelin, although these findings have not been replicated in all published reports [36], [39], [40], [41], [42], [43]. Parenthetically, while the orexigenic effects of acyl-ghrelin are well-established, the biological effects of desacyl-ghrelin – particularly its effects on food intake – have been mixed, ranging from decreased to absent to increased [44], [45], [46]. Nesfatin-1 is another peptide hormone that has been found to be highly co-localized with ghrelin within the stomach [47]. Interestingly, nesfatin-1, which has appetite suppressing actions as well as stimulatory effects on glucagon secretion, also results from prohormone convertase 1/3-mediated processing of its precursor (nucleobindin 2), and is regulated oppositely from ghrelin at the mRNA level in the setting of caloric restriction [40], [47], [48], [49]. The presence of prohormone convertase 2 within gastric ghrelin cells, which unlike prohormone convertase 1/3 is not involved in the processing of proghrelin to ghrelin [23], [37], [50], suggests that other peptide hormones may be present in ghrelin cells.

In the current study, we sought to identify and further characterize other presumed metabolically-relevant proteins secreted by gastric ghrelin cells. Our investigations identified high ghrelin cell expression of both retinol-binding protein (RBP4) and transthyretin (TTR). RBP4 not only transports retinol in the bloodstream, but also has been linked to insulin resistance in both rodent and human models [51], [52]. Transthyretin, previously known as prealbumin and subsequently thyroxine-binding prealbumin, is so named because of its function in transporting both thyroid hormone and retinol binding protein in the bloodstream [53]. We also explored the ghrelin cell-specific secretion and transcriptional regulation of RBP4 and TTR upon caloric restriction and exposure to octanoic acid and β_1_-adrenergic receptor agonists.

## Materials and Methods

### Ethics Statement

All studies were approved by the Institutional Animal Care and Use Committee of UT Southwestern Medical Center (Animal Protocol Numbers 2009-0377 and 2008-0107) and in accordance with the Saitama University Committee on Animal Research.

### Animals

Tissues (stomach, perigonadal white adipose tissue and liver) for quantitative RT-PCR (qPCR) were from 12 wk-old male C57BL/6J mice housed with *ad libitum* access to water and standard rodent diet (Diet 7001; Harlan Teklad, Madison, WI) in a light- and temperature-controlled facility. For certain of the qPCR studies, stomach samples were removed from 10 wk-old male C57BL/6J mice following a 24-hr fast or following 7-days of 60% caloric restriction. For the 60% caloric restriction studies, daily food intake was assessed for each individually-housed study animal during a 3-day run-in period, after which mice were given daily access to only 60% of their usual (average) daily intake. Food restricted mice maintained *ad libitum* access to water. To obtain RNA from ghrelin cell-enriched pools of gastric mucosa cells, 12 wk-old male transgenic ghrelin-hrGFP (humanized *Renilla reniformis* green fluorescent protein) reporter mice (line hrGFP10) were used [23]. For measurement of plasma RBP4, 10 wk-old and 20 wk-old male transgenic mice harboring ghrelinomas (TgGhrelin-SV40-T) were used [25]. For blood collection, mice were deeply anesthetized with an intraperitoneal injection of chloral hydrate (500 mg/kg body weight), after which blood was collected from the inferior vena cava in EDTA-coated tubes containing *p*-hydroxymercuribenzoic acid (final concentration, 1 mM; Sigma-Aldrich, St. Louis, MO). The plasma was separated immediately, acidified with 1N HCl to a final concentration of 0.1 N HCl, and then stored at −80°C until analysis.

### Isolation of ghrelin cell-enriched and non-ghrelin cell-enriched pools of gastric mucosal cells using ghrelin-hrGFP transgenic mice

Separate pools of gastric mucosal cells that were either enriched for ghrelin cells (hrGFP/ghrelin-cell enriched pools) or that mostly lacked ghrelin cells (hrGFP-negative pools) were isolated from transgenic ghrelin-hrGFP mice (line hrGFP10). This process involved a gentle mechanical and enzymatic release of gastric mucosal cells from the stomach followed by cell sorting (MoFlo, Dakocytomation, USA) at the UTSW Medical Center Flow Cytometry Multi-user Core Facility, as described in more detail previously [23], [24], [25], [26]. Four independent fluorescence activated cell sorting (FACS) preparations were included in the subsequent analyses, with 3–5 mice being used for each independent FACS preparation.

### RNA extraction and quantitative RT-PCR

C57BL/6J tissues for qPCR were collected immediately after mice were sacrificed, and were quickly immersed in RNAlater reagent (Qiagen, Hilden, Germany) and then stored at −20°C until extraction. Total RNA was extracted from these tissues using STAT60 (Arcturus Bioscience, Mountain View, CA), according to the manufacturer's protocol. After FACS sorting of hrGFP10 gastric mucosal cells, we adjusted the hrGFP/ghrelin-cell enriched pools and the hrGFP-negative pools to contain a matched number of cells, and the cells in each pool were collected by centrifugation at 4°C at 3000 rpm, for 10 min. Total RNA was extracted from these cellular pellets using STAT60, and was stored at −80°C until use. Total RNA was also extracted from two different mouse ghrelinoma cell lines, SG-1 and PG-1 [25]. Complementary DNA was synthesized from 100 ng total RNA using Superscript III reverse transcriptase (Invitrogen, Carlsbad, CA). The following primer pairs were designed to amplify ghrelin: 5′-GTC CTC ACC ACC AAG ACC AT-3′ and 5′-TGG CTT CTT GGA TTC CTT TC-3′; RBP4: 5′-ACT GGG GTG TAG CCT CCT TT-3′ and 5′-GGA GTA CTG CAG AGC GAA GG-3′; transthyretin: 5′-TGG ACA CCA AAT CGT ACT GG-3′ and 5′-GAT GGT GTA GTG GCG ATG G-3′; nucleobindin 2: 5′-AAA AGC TCC AGA AAG CAG ACA-3′ and 5′-GCT CAT CCA GTC TCG TCC TC-3′; cyclophilin: 5′-TGG AGA GCA CCA AGA CAG ACA-3′ and 5′-TGC CGG AGT CGA CAA TGA T-3′. Messenger RNA levels were measured with an ABI 7300 Real-Time PCR System (Applied Biosystems, Foster, City, CA). Quantitative RT-PCR was performed using the iTaq SYBR Supermix (Bio-Rad Laboratories, Hercules, CA), according to the manufacturer's instructions. Initial template denaturation (3 min at 95°C) was performed, followed by 40 cycles of denaturation (15 sec at 95°C) and annealing/extension (45 sec at 60°C). Reactions were evaluated by the comparative threshold cycle (C_T_) method, using cyclophilin as the invariant control gene. Previously, we have reported comparing the C_T_ values of several genes amplified from FACS-separated gastric mucosal cells with the C_T_ values of a separate housekeeping gene, 36B4, and observed results similar to those determined with cyclophilin [24]. The efficiencies of the primers were validated by verifying a slope of approximately −3.3 when the logs of the cDNA concentration at different serial dilutions were compared with the C_T_. The primers were designed to amplify regions of cDNA that in the corresponding genomic DNA span introns to ensure the amplification of cDNA derived from mRNA rather than residual genomic DNA.

### Gene Chip Array Analysis

After FACS separation of hrGFP10 gastric mucosal cells, we adjusted the hrGFP/ghrelin-cell enriched pools and the hrGFP-negative pools to contain a matched number of cells (15,000 cells), and the cells in each pool were collected by centrifugation at 4°C at 3000 rpm, for 10 min. Total RNA was extracted from the cells using the Pico Pure RNA isolation kit (Arcturus Bioscience). Fifty ng total RNA was used for making labeled cRNA with the Affymetrix Two-Cycle Target Labeling Protocol (Affymetrix, Santa Clara, CA). Labeled cRNA was hybridized to Affymetrix Mouse Genome 430 2.0 arrays. Hybridization, washing and scanning were carried out by the UTSW Microarray Core Facility. Data was analyzed with GeneChip Operating Software version 1.0 using the mas5 algorithm.

### In vitro secretion studies

Cells from the immortalized ghrelin cell line PG-1 were used for these studies [25]. On day 0, PG-1 cells were plated in DMEM/F-12 50∶50 medium (Mediatech, Inc., Manassas, VA) containing 10% (vol/vol) fetal bovine serum (FBS) and supplemented with 50 µM octanoate-BSA, 100 U/mL penicillin and100 µg/mL streptomycin sulfate. The cells were placed into the wells of poly-L-lysine-coated 24-well plate culture dishes and incubated in humidified 95% air and 5% CO_2_ at 37°C. Cells were maintained in DMEM/F-12 with 10% FBS in a 37°C incubator with 5% CO_2_. On day 2, the media was aspirated and replaced with serum-free DMEM containing 5.5 mM glucose, 100 U/mL penicillin and 100 µg/mL streptomycin sulfate with different concentrations of octanoate-BSA. Alternatively on day 2, the media was aspirated and replaced with serum-free DMEM containing 5.5 mM glucose, 100 U/mL penicillin and 100 µg/mL streptomycin sulfate and 50 µM octanoate-BSA containing either 10 µM epinephrine or 10 µM norepinephrine (Sigma-Aldrich). The day 2 incubations were performed for 6 hours in humidified 95% air and 5% CO_2_ at 37°C, and then the media was immediately collected and centrifuged at 3,000× g for 5 min. The supernatant was stored at −80°C until analysis. These culture conditions are slightly different than those previously reported, however these slight modifications did not alter the previously-reported ghrelin secretory response of the cells to norepinephrine [25].

### Detection of RBP4 protein by ELISA and Western Blot analysis

RBP4 protein levels were determined using RBP4 (mouse/rat) Dual ELISA kit (Cat. No. AG-45A-0012EK-KI01, Adipogen, Incheon, Korea), according to the manufacturer's instructions. In addition, the presence of RBP4 protein was determined using Western Blot analysis. To prepare samples, SG-1 cells were cultured with DMEM/F-12 with 10% FBS in a 37°C incubator with 5% CO_2_ and 5% O_2_ for 2 days, after which the cells and culture media were collected together and centrifuged at 1200× rpm for 5 min. The supernatant (culture media) was separated from the cell pellet and stored at −80°C until protein measurement. The cell pellet was washed twice with cold PBS, after which 1 mL RIPA buffer (Nacalai Tesque, Inc., Kyoto, Japan) including 1× PMSF (Sigma) and 1× protease inhibitor cocktails (Nacalai Tesque) was added. The cells were homogenized for 10 min while on ice, and the resulting cell lysate was centrifuged at 12,000× g for 10 min. The supernatant (cell lysate) was stored at −80°C until protein measurement. Total protein concentrations of the culture media and cell lysate samples were measured by BCA™ Protein Assay kit (Thermo, Rockford, USA) according to the manufacturer's instructions. Equal amounts of protein (45 µg/well for cell lysate, 17 µg/well for medium) were run on 12% sodium dodecyl sulfate-polyacrylamide gels and electroblotted onto PVDF membranes. The membranes were probed successively with primary anti-human Retinol-Binding Protein antisera (Lot:00066392, Dako, Glostrup, Denmark) diluted 1∶1000 and then HRP-labeled secondary antibody (Promega, Madison, WI, USA) diluted in 1∶100,000, for 2 h at room temperature. Specific antigen-antibody binding was visualized with LuminataTM Forta Western HRP substrate (Millipore Corporation, Billerica, USA) using a ChemiDoc XRS System (Bio Rad, Hercules, CA, USA).

### Ghrelin measurements

Acyl-ghrelin concentrations were determined using a Rat acylated ghrelin enzyme immunoassay kit (#A05117, Bertin pharma, Montigny le Bretonneux, France) according to the manufacturer's guidelines. Samples were diluted 1∶ 5 for the measurements.

### Statistical analysis

Data are expressed as mean ± standard error of the mean (SEM). GraphPad Prism 5.0 (GraphPad Software, Inc., San Diego, CA) was used for statistical analyses. For Table 2 qPCR studies, one-way ANOVA followed by Dunnett's post hoc test was used to compare levels of mRNA in each tissue to whole stomach; in cases of high variance, log transformation of data was performed prior to statictical analysis. For all other analyses, one-way ANOVA followed by Tukey's post hoc test was used and *P*<0.05 was considered to be statistically significant.

**Table 2:** Relative levels of mRNAs in various mouse tissues and mouse ghrelinoma cell lines.

mRNA	whole stomach	hrGFP-negative pools	hrGFP/ghrelin cell-enriched pools	SG-1 ghrelinoma cell line	PG-1 ghrelinoma cell line	liver	white adipose tissue
ghrelin	1.0	0.03±	1989±	103.0±	56.2±	0.0003±	0.0005±
	(15.95±0.5)†	0.02*	292*	12.6*	13.0*	0.0001*	0.0001*
RBP4	1.0	0.3±	75.9±	249.8±	128.1±	244.6±	80.0±
	(21.8±0.6)	0.2*	11.8*	58.3*	17.6*	14.9*	4.7*
TTR	1.0	0.9±	151.2±	281.2±	473.3±	242.9±	0.004±
	(22.0±0.3)	0.3	71.7*	61.3*	52.9*	62.0*	0.001*
NUCB2	1.0	0.09±	2.9±	3.1±	1.5±	0.07±	6.4±
	(22.4±0.3)	0.04*	0.8	0.5*	0.1	0.001*	0.6*

Each value represents the amount of mRNA relative to that in whole stomach, which is arbitrarily defined at 1.0 and is shown as the means ± SEM of 3 different preparations. Each determination was done in duplicate.

† Values in parentheses denote the mean ± SEM of threshold cycle values.

* Level significantly different from whole stomach (*P*<0.05).

## Results

### Expression of RBP4 and transthyretin (TTR) mRNA in ghrelin cells

In an effort to broadly identify proteins expressed highly within ghrelin cells, we screened a gene chip array that had been prepared from RNA isolated from pools of gastric mucosal cells highly enriched for ghrelin cells (hrGFP/ghrelin-cell enriched pools). Data from this gene chip were compared to those generated using RNA isolated from pools of gastric mucosal cells from which ghrelin cells had been mostly removed (hrGFP-negative pools). Separation of the different gastric mucosal pools was achieved by fluorescence activated cell sorting of gastric mucosal cells isolated from transgenic ghrelin-hrGFP reporter mice, as done previously [23], [24], [25], [26]. A selected list of molecules identified using this method is included in Table 1. As expected, signal intensities reflecting numbers of ghrelin and ghrelin O-acyltransferase (GOAT) mRNA transcripts, were markedly higher on the gene chip probed with mRNA from hrGFP/ghrelin cell-enriched pools as compared to the gene chip probed with mRNA from hrGFP-negative pools. Messenger RNA for serum retinol binding protein 4 (RBP4), which is of interest due to the known association of its encoded protein with disorded metabolic states, also was present at relatively high levels in the hrGFP/ghrelin cell-enriched pools. Because RBP4 is known to circulate in the bloodstream bound to transthyretin (TTR), we also searched the gene chip for TTR mRNA and found its signal intensity to be increased in the hrGFP/ghrelin cell-enriched pools. For comparison, we had previously identified both chromogranin A, prohormone convertase 1/3, and prohormone convertase 2 mRNA expression within ghrelin cells [23], and these again were identified as being present at relatively high levels in the hrGFP/ghrelin cell-enriched pools. As expected from previous ghrelin co-localization studies, nucleobindin 2 (which encodes nesfatin-1) and regulated endocrine-specific protein 18 (RESP18), an endoplasmic reticulum-localized protein of unclear physiological function, also both were found at high levels in the hrGFP/ghrelin cell-enriched pools [47], [54].

**Table 1:** Relative levels of selected set of mRNAs within FACS-separated pools of gastric mucosal cells, as determined by Affymetrix array.

mRNA	hrGFP/ghrelin cell-enriched pools signal intensity	hrGFP-negative pools signal intensity	fold-increase
ghrelin	47159	159	137
GOAT	25977	142	169
RBP4	8769	276	42
TTR	8681	543	21
chromogranin A	38699	2679	8
prohormone convertase 1/3	3564	342	11
prohormone convertase 2	3962	55	79
NUCB2*	11544	2168	6
RESP18	19795	82	239

* NUCB2 = nucleobindin 2, which encodes nesfatin-1.

To independently confirm the expression data revealed by the gene chip arrays, quantitative RT-PCR analysis on several tissue samples was performed using specific oligonucelotide primers (Table 2). Amplification of complementary DNA prepared from whole stomach and FACS-separated gastric mucosal cells from ghrelin-hrGFP mouse stomachs demonstrated significantly higher ghrelin mRNA levels in the hrGFP/ghrelin cell-enriched pools and in two different immortalized ghrelin cell lines derived from transgenic mice harboring SV40 large T-antigen-induced ghrelinomas (SG-1 and PG-1) as compared to whole stomach, as demonstrated previously [25]. Similarly, as compared to their mRNA expression levels within whole stomach, RBP4 and TTR levels within highly enriched pools of FACS-separated gastric ghrelin cells were substantial, as were RBP4 and TTR levels within the ghrelinoma cell lines. As had been observed previously for ghrelin transcripts and other transcripts within SG-1 and PG-1 cell lines, levels of RBP4 and TTR transcripts within the ghrleinoma cell lines did not match exactly those observed in the FACS-separated gastric ghrelin cells [25], [26]. In contrast, ghrelin mRNA was barely detected by qPCR in liver and white adipose tissue, while, as expected from previous work, RBP4 mRNA was detected in both liver and white adipose tissue, and TTR mRNA was high in liver but not white adiopose tissue [53], [55]. The mean RBP4 mRNA level in the hrGFP/ghrelin cell-enriched pool approached 31% of that in liver and was nearly 95% of that observed in WAT. The mean TTR mRNA within the hrGFP-positive pool was even closer (62%) to that within the liver.

### Expression of nucleobindin 2 mRNA in ghrelin cells

Previous work has demonstrated expression of nucleobindin 2 (NUCB2) mRNA and one of its gene products, nesfatin-1, in ghrelin cells [47]. Thus, for comparison, we also analyzed NUCB2 mRNA levels using the same qPCR strategy as above. Here, NUCB2 mRNA was detected by qPCR within the hrGFP/ghrelin cell-enriched pools, although levels were not significantly higher than in whole stomach (Table 2). Presumably this lack of enrichment is due, at least in part, to expression of NUCB2 in other gastric mucosal cells such as histamine-containing and somatostatin-containing cells in which it also was previously identified [47]. As compared to RBP4 and TTR, the relative amounts of NUCB2 mRNA within the hrGFP/ghrelin cell-enriched pools were lower.

### Regulation of RBP4 and TTR mRNA expression in whole stomach by caloric restriction

**Figure 1:**
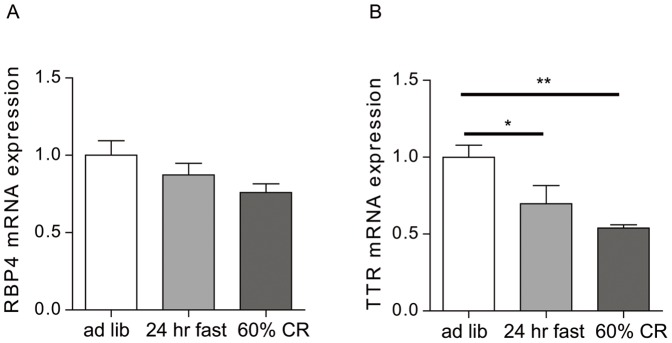
Effects of caloric restriction on RBP4 and TTR mRNA levels in the stomach. Relative RBP4 (A) and TTR (B) mRNA levels within the stomachs of ad lib-fed, 24-hr-fasted and chronically food restricted [60% of usual daily calories for 7 days (60% CR)] C57BL/6J mice were determined using qPCR. Statistically significant effects of the two caloric restriction protocols were noted only for TTR (*P<0.05 and **P<0.01).

As ghrelin transcription within and secretion from ghrelin cells is known to be regulated by changes in metabolic state, we next examined changes in RBP4 and TTR mRNA expression within the whole stomach upon caloric restriction, using a more acute model (24-hour fast) and a more chronic model (daily exposure to only 60% of usual daily calories, for 7 days). In neither the acute nor chronic caloric restriction model were statistically significant changes to RBP4 mRNA observed (Fig. 1A). On the other hand, TTR mRNA expression within the stomach was significantly decreased upon a 24-hour fast and 60% calorie restriction, to nearly 70% and 54% the levels observed in stomachs from *ad lib*-fed mice, respectively (Figure 1B).

### RBP4 secretion from immortalized ghrelin cell lines

**Figure 2:**
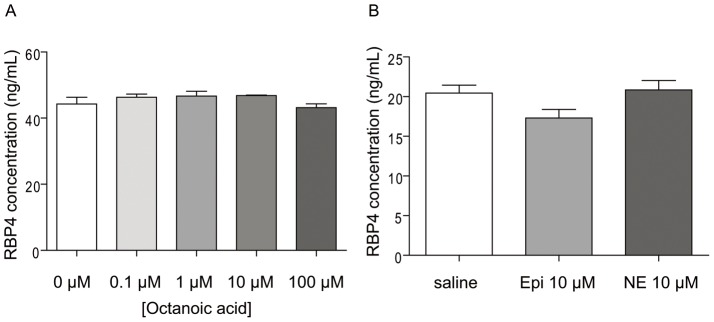
The effect of octanoic acid, epinephrine and norepinephrine on RBP4 release from immortalized ghrelin cells. RBP4 protein levels were measured by ELISA in the culture media of PG-1 cells incubated for 6 hrs in the presence of different concentrations of octanoic acid, 10 µM epinephrine (Epi) or 10 µM norepinephrine (NE).

Next, we examined secretion of RBP4 from the PG-1 immortalized ghrelin cell line [25]. Previously, both this PG-1 line and a second ghrelinoma cell line (SG-1) were shown to serve as good models with which to study mechanisms of ghrelin release, demonstrating similar gene expression profiles as well as similar responses to candidate ghrelin secretagogues [25]. In particular, in prior work, exposures of PG-1 cells and SG-1 cells to octanoic acid, epinephrine and norepinephrine were each shown to dose-dependently enhance secretion of acyl-ghrelin; epinephrine and norepinephrine also stimulated desacyl-ghrelin release [25]. Here, although we detected RBP4 by ELISA assay in the culture media of PG-1 cells, neither octanoic acid, using doses ranging from 0.1 µM to 100 µM, 10 µM epinephrine nor 10 µM norepinephrine, altered levels of RBP4 in the media upon a 6-hour exposure (Fig. 2).

**Figure 3:**
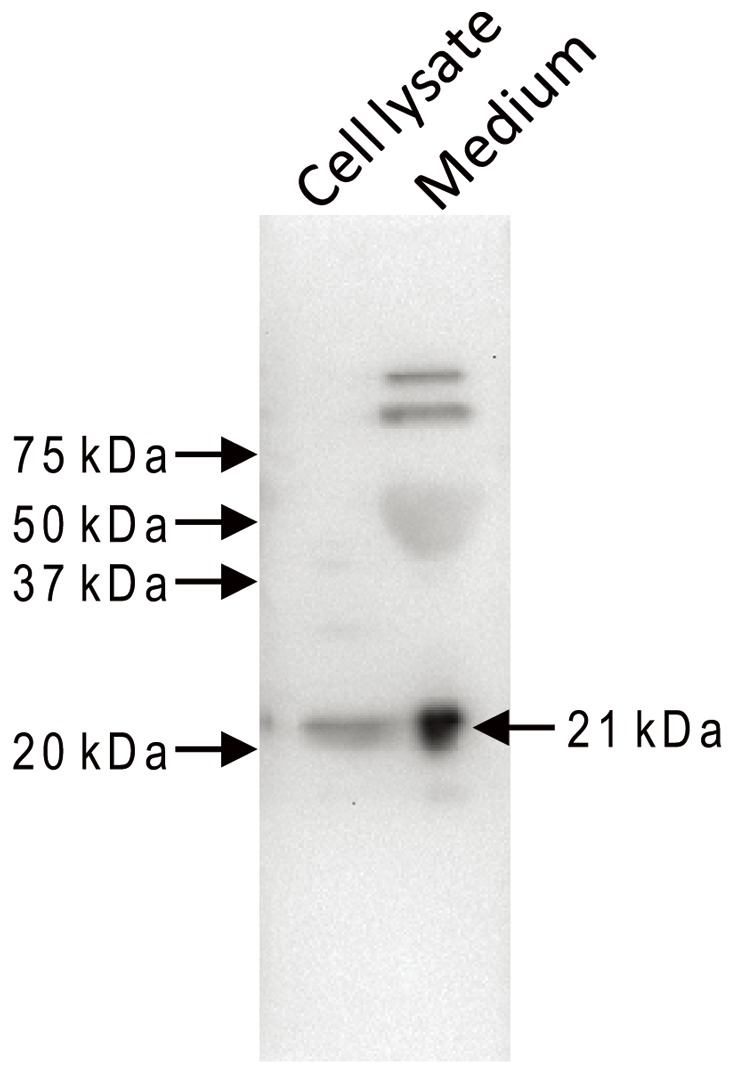
Expression of RBP4 by immortalized ghrelin cells. A 21-kDa band corresponding to the expected size of RBP4 protein was detected in SG-1 cell lysate and SG-1 culture media by Western blot analysis using anti human RBP4 antisera. A representative blot is depicted.

Similarly, RBP4 protein was detected in both SG-1 cell culture media and SG-1 cell lysates by Western blot analysis (Fig. 3). However, neither a similar range of octanoic acid doses nor 10 µM norepinephrine was able to stimulate RBP4 release from SG-1 cells, despite the ability of the 10 µM norepinephrine to stimulate ghrelin release into the culture media, as had been demonstrated previously [25] (data not shown). Lack of a similar pattern of stimulated RBP4 and ghrelin secretion by octanoic acid and catecholamines suggests that RBP4 and ghrelin cellular localization and secretion regulatory mechanisms also are not similar.

### Plasma RBP4 levels in mice harboring ghrelinomas

**Figure 4:**
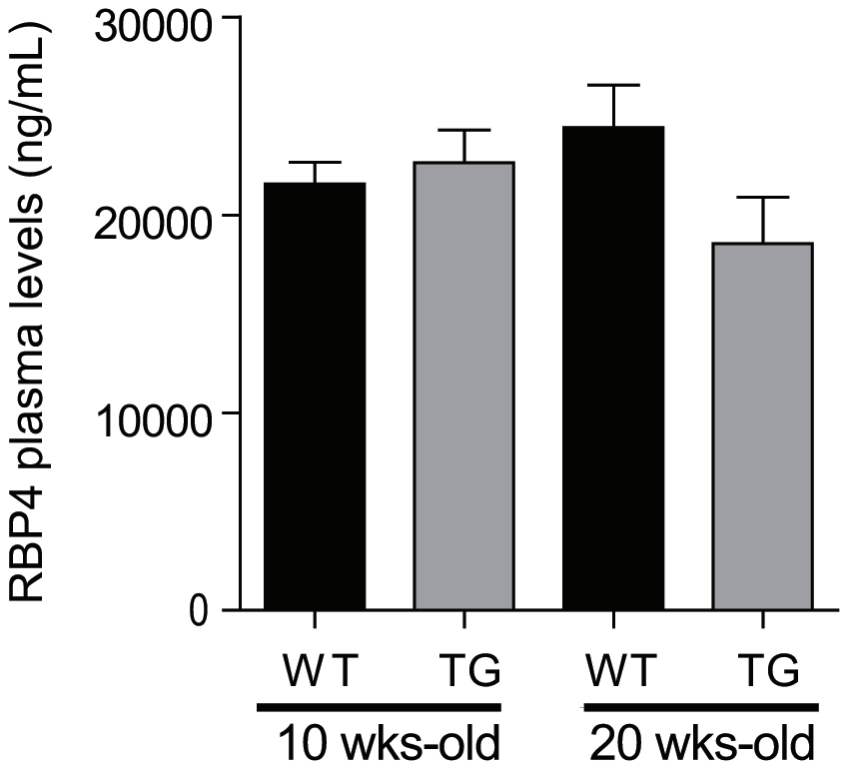
Plasma RBP4 concentration in ghrelin-SV40 T-antigen transgenic mice. Plasma concentration of RBP4 in wild type (WT) and ghrelin-SV40 T-antigen transgenic (TG) mice at 10 and 20 wks of age, as measured by ELISA.

Next, we sought to determine if expanded ghrelin cell numbers could influence circulating levels of RBP4 *in vivo*. To accomplish this, plasma RBP4 levels were measured using the same transgenic mouse line used to derive the PG-1 and SG-1 ghrelinoma cell lines, in transgenic mice and wild-type littermates aged 10 wks and 20 wks. Previously, we had demonstrated approximately 2-fold increases in acyl-ghrelin and desacyl-ghrelin plasma levels in 10 wk-old mice harboring the ghrelin-SV40 T-antigen transgene as compared to 10 wk-old wild-type littermates; a 25-fold increase in plasma acyl-ghrelin and a 37-fold increase in plasma desacyl-ghrelin were noted in 20 wk-old transgenic mice [25]. The marked increase in ghrelin in the 20 wk-old mice has been presumed to correspond with the SV40 T-antigen-driven clonal expansion of ghrelin cells [25]. Of note, for most of the mice harboring the ghrelin-SV40 T-antigen transgene, noticeable abdominal cavity tumors do not become apparent in live animals until the age of 24 wks, although the elevated plasma ghrelin in younger mice suggests the presence of hyperplastic ghrelin cells at those earlier ages [25]. Here, despite the expanded mass of ghrelin cells, no statistically significant increase in plasma RBP4 levels was apparent in either 10 wk-old mice or 20 wk-old mice (Fig. 4).

## Discussion

In this paper, we describe for the first time marked expression within gastric ghrelin cells of the secreted proteins RBP4 and TTR. For RBP4, this marked expression was first suggested at the mRNA level in FACS-sorted hrGFP-positive cells from ghrelin-hrGFP reporter mice, using gene chip technology. Such was confirmed by qPCR studies using not only the hrGFP/ghrelin cell-enriched samples but also two different mouse ghrelinoma cell lines. Similar to the observed enrichment of RBP4 noted in FACS-sorted hrGFP-positive cells, mRNA for TTR, which serves as a transport protein for both RBP4 and thyroid hormone, also was expressed within the stomach, where it was highly enriched within the ghrelin cell population. Although RBP4 was found in lysates and the media of cultured ghrelinoma cells, we did not observe octanoic acid or catecholamine stimulation of RBP4 secretion, as had been observed previously when the same conditions were used to assess acyl-ghrelin secretion [25]. Nor did we observe changes in RBP4 mRNA levels in whole stomach upon caloric restriction. We also did not observe any statistically significant increase in plasma RBP4 in mice harboring ghrelinomas. Caloric restriction did, however, decrease TTR mRNA in whole stomach.

The significance of these findings stems not only from the known biological functions of RBP4 and TTR, but also from the mostly uncharacterized physiology of the ghrelin cell. RBP4 is a 21-kDa protein synthesized by and secreted mainly from the liver [55]. Its best characterized function is to deliver retinol (vitamin A) from hepatic retinoid stores to tissues throughout the body, where it is metabolized to retinaldehyde and retinoic acid [56]. More specifically, RBP4 is the only specific serum transport protein for retinol and thus serves an important function for a multitude of retinol-dependent functions, including vision, growth and development and immune regulation [56], [57].

More recently, plasma RBP4 has been identified as an important signal of insulin resistance [57]. This first became evident in insulin resistant mice lacking GLUT4 (glucose transporter 4) specifically in white adipose tissue, in which serum RBP4 was markedly elevated as was the level of white adipose tissue RBP4 mRNA [52]. Previously in rodents, adipose tissue had been reported to contain relatively high levels of RPB4 mRNA, averaging between 6–37% those in liver [58], [59]. Circulating RBP4 is also elevated in several other mouse models of insulin resistance as well as in insulin-resistant humans [51], [52]. Interestingly, pharmacologic administration of RBP4 and transgenic overexpression of RBP4 both induce insulin resistance in mice, while genetic deletion of RBP4 enhances insulin sensitivity [52]. These findings and others suggest that elevated serum RBP4 may play a causative role in the development of insulin resistance, although it is as yet unclear if this involves retinol-dependent mechanisms, retinol-independent mechanisms, or both [57].

Retinol-RBP4 circulates in the bloodstream in a 1∶1 molar complex with TTR [60], [61]. Like RBP4, TTR also is synthesized mainly in the liver [53]. Not only does TTR transport the retinol-RBP4 complex in the bloodstream, but also it prevents glomerular filtration of RBP4 at the kidney, thus helping to prevent loss of retinol and RBP4 in the urine [62]. Regarding its role in thyroid hormone transport in the bloodstream, in humans, TTR carries about 10–15% of T_4_ and T_3_ (as opposed to thyroxine binding globulin, which carries about 70%, albumin, which carries about 15–20%, and lipoproteins, which carry a fraction), although its relatively low affinity for T4 and T3 leads to a more immediate delivery of thyroid hormone to target tissues [63]. In mice, TTR serves as the major thyroid hormone transport protein [63]. TTR also is synthesized in the choroid plexus, which likely accounts for its presence in cerebrospinal fluid [63]. Within the CSF of both humans and mice, TTR functions as the major carrier of thyroid hormone; in this role, TTR is thought to facilitate the passage of thyroid hormone across the choroid plexus and then distribute it to the central nervous system [63].

Of interest, RBP4 and TTR expression have been localized to more sites than just the liver plus white adipose tissue (RBP4) and the liver plus choroid plexus (TTR). Using Northern Blot analysis on various rat tissues, RBP4 mRNA has been detected in the kidney at 5–10% of hepatic levels, in lung, spleen, brain, heart and skeletal muscle at 1–3% of hepatic levels, and in the intestine, testis and pancreas at <1% of hepatic levels [64]. Other studies have shown RBP mRNA in rat eye, where it is localized to the retinal pigment epithelium, and in the visceral yolk sac [64], [65]. As it relates to the current study, Northern blot analysis also has revealed RBP4 mRNA levels in stomach at 1–3% of hepatic levels [64]. Here, using qPCR, RBP4 mRNA levels within whole stomach reached only 0.4% of those in the liver while RBP4 mRNA levels within the hrGFP/ghrelin cell-enriched pools reached 31% of those in the liver. Thus, the current data suggests that the RBP4 expression within ghrelin cells likely accounts for the majority of its stomach expression. Like RBP4, TTR is also synthesized in the rat pigment epithelium [66]. Additionally, it is produced in the placenta and in the islets of Langerhans [67], [68]. TTR expression within the gastrointestinal tract also has been described previously, although never before co-localized with ghrelin as described in the current study. In particular, transgenic mice in which Cre recombinase or red fluorescence protein expression are directed by TTR promoter elements manifest reporter activity in the gastric mucosa of E12.5–E16.5 embryonic animals [69]. Here, in adult mice, TTR expression in the stomach approached 0.4% of its levels in liver, while that in hrGFP/ghrelin cell-enriched pools approached 62% of its levels in liver.

It has been proposed that the sites of RBP4 and TTR biosynthesis may help dictate their functions. In a study specifically designed to investigate the physiological role of circulating RBP4 produced outside the liver, handling of retinol was examined in mice in which endogenous RBP4 expression was genetically deleted and replaced with RBP4 overexpressed in muscle. Unlike liver-derived RBP4, the extrahepatically synthesized RBP4 failed to mobilize liver retinol stores in this mouse model [61]. This led the authors of that study to postulate that RBP4 “has a function specific to its tissue of origin” [56], [61]. Supporting this statement, it has been suggested that RBP4 and TTR made in the visceral yolk sac may be involved in transport of retinol and thyroid hormone from the maternal circulation to the developing fetus [53]. As mentioned, choroid plexus-derived TTR is thought to mediate the distribution of thyroid hormone to the central nervous system, and furthermore, also may help protect against ischemia-induced brain injury [63], [70]. In the case of islet-derived TTR, it has been theorized that the protein could affect the processing of glucagon, and furthermore that it may influence deposition of islet amyloid, which is found in at least 95% of subjects with Type II diabetes mellitus [71].

An as-of-yet unanswered question raised by the current study is why RBP4 and TTR are expressed within ghrelin cells, especially at such high relative levels. One might postulate that their expression is related to the emerging role of ghrelin cell-derived products, and thus, the ghrelin cell as a whole, in metabolism. As mentioned, acyl-ghrelin, desacyl-ghrelin, obestatin, nesfatin-1 and RBP4 all have been shown (albeit in certain cases, inconsistently) to affect food intake, body weight and/or blood glucose handling. Relationships of RBP4 to some of these other ghrelin cell products has been examined in at least one human study, in which 6 mo following gastric band surgery, which resulted in a reduction of BMI of on average 6 kg/m^2^, circulating levels of total ghrelin increased, obestatin increased and RBP4 decreased [72]. Whereas low circulating ghrelin has been associated with elevated fasting insulin and insulin resistance in humans [73], [74], the opposite is true for RBP4 [51]. Here, regulation of TTR mRNA expression in the stomach by caloric restriction also suggests that ghrelin-cell derived TTR may be involved in metabolism.

Despite the high level of RBP4 mRNA within ghrelin cells, especially as compared to liver and white adipose tissue, its overall contribution to the complement of RBP4 circulating in the bloodstream is seemingly low. The stomach is a relatively small organ, especially as compared to the liver and white adipose tissue, and furthermore, the ghrelin cell population is limited to approximately 0.3–1% cells comprising the entire gastric mucosa [23]. Even with the marked expansion of ghrelin cells in the mice harboring ghrelinomas, total circulating RBP4 level remained unchanged. This perhaps suggests that ghrelin cell-derived RBP4 and TTR may not impact whole body insulin resistance and/or thyroid hormone transport. Rather, ghrelin cell-derived RBP4 and TTR instead may have effects that are more localized to the stomach, just as has been suggested for other tissues. Although co-expression of RBP4 with TTR within the ghrelin cell does suggest that there exists a shared function, the roles of ghrelin cell-derived RBP4 and TTR could be unrelated.

In conclusion, we have found relatively high levels RBP4 and TTR expression by gastric ghrelin cells. Thus, RBP4 and TTR join a short list of other proteins known to be secreted by ghrelin cells, including acyl-ghrelin, desacyl-ghrelin, obestatin and nesfatin-1. At this time, the etiology of their expression within ghrelin cells can only be speculated, although it is assumed that this could be related to an emerging general role for the ghrelin cell in blood glucose handling and metabolism. Alternatively, RBP4 and TTR may have a more regional function unique to ghrelin cells and their neighboring cells. Future studies in which RBP4 and/or TTR expression can be deleted specifically from ghrelin cells may be helpful in pinpointing the actions of ghrelin cell-derived RBP4 and TTR.
